# Round the Hospitals

**Published:** 1920-01-31

**Authors:** 


### ROUND THE HOSPITALS.

THE KING has commanded on the recommendation of his Secretary of State for War that the following appointments to the Order of the British Empire, published in the *Gazette* on the days stated, shall be transferred to the Military Division of the Order: Miss M. S. Milne, late matron Q.A.I.M.N.S.R. (June 7, 1918); Miss M. E. Todd, matron Q.A.I.M.N.S. (June 7, 1918).

THE Royal Red Cross, First Class, has been awarded by the King to the following ladies in recognition of valuable nursing services under the British Red Cross Society and the Order of St. John of Jerusalem. (Final List). Miss Davies, A.R.R.C., Matron Royal National Hospital, Ventnor; Mrs. K. L. Guise-Moores, A.R.R.C.; Sister L. Hicks; Miss F. E. Hunt, A.R.R.C.; Miss

**ROUND THE HOSPITALS**—(*continued*).

M. E. Johnston, A.R.R.C.; Miss M. Kempton, Matron Royal Sea Bathing Infirmary, Margate; Miss I. C. Mackintosh, A.R.R.C.; Miss M. B. Monk. A.R.R.C., Matron of the London Hospital; Miss M. H. Prys-Owen; Mrs. F. H. Stephens, A.R.R.C.; Miss G. A. B. Stevens, A.R.R.C.; Mrs. K. H. E. Talbot, A.R.R.C. The King has also awarded the Royal Red Cross, Second Class, to a large number of matrons, sisters, nurses and members of Voluntary Aid Detachments, mainly for services in connection with auxiliary hospitals.

---

EARNEST appeals are made by Princess Christian, Chairman of the British Committee of the Russian Red Cross, for the wounded and suffering in Russia. This Committee deals with the supply of drugs and medical stores to combat disease, and its resources are being taxed to the uttermost to cope with the spread of typhus, smallpox and other diseases which are the outcome of privation. Besides this there is a special committee to administer relief to the Russian civilians and refugees in Great Britain. Workrooms have been established at 21 Belgrave Square, where voluntary helpers are needed, and where refugees, many belonging to the educated classes, are employed in making clothing for the destitute areas, while their children are fed and looked after. A relief fund with headquarters at 30 Bedford Square provides small pensions for Russian refugees too old or infirm to work. Masses of refugees are still flying before the advancing Bolshevik armies, and are in a destitute state. Gifts in kind can be sent to the Committee's stores at 167 Waterloo Road, S.E. 1; gifts of money will be gratefully received by the Secretary of the Russian Red Cross (British Committee), 35 Albemarle Street, E.C. 1.

---

MORE accommodation for the out-patient department and the medical school were described by Sir G. H. Makins as the chief needs of St. Thomas's, in proposing the toast of that hospital at the Old Students' Dinner. He also mentioned that the hospital governors were willing to find room on a new site adjoining the hospital for a club which should serve as a memorial of those St. Thomas's men who served in the war, and for a residential portion to be used by former students visiting London. Dr. Addison was present at the dinner. He expressed his belief that means could be found whereby the great voluntary hospitals—the leader in medical matters for generations—could help and receive help from the State without having their machinery or character as voluntary institutions destroyed. "National assistance given to medical schools," he said, "need not interfere with or check their progress; but, in return, the State would look to such organisations for increased assistance in training men for specialised medical services.

---

THE *Daily Telegraph* Shilling Fund in aid of the endowment of the College of Nursing and the Nation's Fund for Nurses opened auspiciously on January 26 with donations of 1,000 shillings from the Bishop of London, from the promoters of the Treloar Home, and other persons specially connected with the work of nurses. That the Fund will elicit a wide response from the public is a foregone conclusion. It appeals to an ever-widening circle of the public who have personal reasons for feeling gratitude towards nurses, and offers an unequalled opportunity for the bestowal of small as well as large donations. All persons concerned to redeem from want the ailing, anxious, or aged members of the profession are stirred to gratitude by Lord Burnham's decision to reinforce the splendid efforts of the British Women's Hospital Committee, and to start the list with 10,000 shillings as the *Daily Telegraph's* own subscription. Probably few men, if any, have done more work for the nation and everybody since the war began than Lord Burnham, whose generosity and kindness always are as remarkable as they are grateful to encounter.

---

IN addition to the practical financial results of the *Daily Telegraph* Shilling Fund, the nursing profession stands to gain enormously by the clear communication in an influential daily paper of the purposes which animate the modern nursing profession. Contributions are invited, not solely to secure from want nurses who have suffered under the hardships entailed by their calling, but also to endow the College of Nursing, and render it a great engine for the amelioration of the profession. The educational aims of the College occupy a prominent place among the causes which have led to this appeal to the public. Without depreciating the value which attaches to State or municipal aid in building up great educational centres, it must never be forgotten that such centres as Oxford and Cambridge, such foundations as the Royal Colleges of Physicians and Surgeons, owe their inspiration and influence to the munificence of the men and women who endowed them in the past. The *Daily Telegraph* tells the public plainly that the cause of the nurses ought not to rest solely on a small group of wealthy philanthropists. It is the cause of the whole British nation, and the endowment should be furnished by all who have cause to bless their ministrations.

---

BIRMINGHAM citizens, keenly appreciative of the benefits conferred on the children of the city by the Princess Alice Orphanage (National Children's Homes), met a few days ago under the Presidency of the Lord Mayor to keep the annual festival of the homes and inspire each other to the task of keeping them not only going but growing. The Principal of the Homes, the Rev. W. Hodgson Smith, recorded the unparalleled progress and unparalleled difficulties of the past year. The waiting-list of children is 200 long, but they have lately been presented with a fine country mansion in Gloucestershire with eight acres of ground attached which will take in from sixty to seventy more children. The immediate task before them is to raise £15,000, or better still £20,000, to set their finances on a sound basis. There is a great increase

**ROUND THE HOSPITALS**—(*continued*).

in the demand for orphanage accommodation, due directly and indirectly to the war, and judging from the past, Birmingham is not going to allow any of its children to remain uncared for.

Building operations in connection with the Rye Memorial Hospital will shortly commence. The generous donors, Lady McIlwraith and her son-in-law and daughter, Mr. and Mrs. McGowan, who gave the Fund a splendid start, have now, we understand, shouldered entire responsibility for the cost of erecting a hospital of from six to ten beds, leaving the balance of other subscriptions after the equipment has been provided to form an endowment. The immediate need for the hospital is shown by the fate which recently befell a female patient in one of the outlying districts near Rye. She was removed to Hastings to undergo an operation at the East Sussex Hospital, but could not be received there owing to lack of accommodation and has now returned home in a critical state. The medical officers of the district attach great importance to the installation of an *x*-ray department, recourse to which is now indispensable in general practice, and have had the promise of the necessary equipment.

The new Maternity Hostel at Canterbury was opened on January 12 by the Archbishop of Canterbury, attended by a large company, including Mrs. Randall Davidson and the Mayor and Mayoress. The new building is very pleasantly situated on the Dane John. It will take in six ordinary, in addition to two paying, patients, and can train three midwifery pupils. The first matron is Miss Maycock, who will be assisted by Sister Bridger and another nurse. It is believed that when fully in working order it will, with the aid of a grant from the Ministry of Health, be self-supporting. The Archbishop spoke of the worthy traditions of the Maternity Association, dating back 119 years, which has carried out this enterprise, and of the sanctity of maternity work, and appealed for further support in gifts of all kinds, as well as money, to give the hostel a good start.

Grave difficulties are being experienced in meeting the need for district nurses in Cornwall. It is not that Cornish women are backward in applying for training. It is that, once launched on their career, they are apt to succumb to the temptation afforded by better appointments elsewhere. And why, indeed, should they not? Nurse-midwives seem to be particularly badly off in Cornwall. In Devon the County Council makes a payment of from £10 to £20 for each nurse employed, on condition that a minimum salary (amounting usually to £100) is given by the local Nursing Association. In Cornwall the minimum salary is £80. At the recent meeting of the Sanitary Committee of Cornwall a report was read and adopted from a sub-committee appointed to investigate these matters, and it was resolved that the pay of Cornish nurse-midwives should be raised to a minimum of £100, and that the Sanitary Committee should make themselves responsible for the payment of a subsidy of £15 per nurse wherever the salary did not fall below £100. After a year's trial of this plan the matter will be reconsidered.

Among the occupations open to disabled nurses none is more closely connected with their previous interests and more interesting in character than radiography. A six months' course on this subject is now given to male or female students in many leading hospitals. It embraces (1) the taking, developing, and printing of *x*-ray photographs of radiograms and a thorough working and repairing knowledge of the apparatus used; (2) the elements of electricity, anatomy, and pathology; (3) some acquaintance with actino-therapeutics or the treatment of skin diseases. There is already a considerable demand for operators trained to exact methods and possessed of the requisite pitch of accuracy, and this is likely to develop as one after another the minor institutions of the country are finding an *x*-ray installation indispensable. A knowledge of nursing is a distinct asset in this calling.

Nursing opinion in South Africa appears to be pretty comprehensively ignored by the legislators who are making laws to regulate their profession. The Transvaal Provincial Council has issued an Ordinance providing for the registration and part inspection of private nursing homes, but for want of appreciating the nursing point of view they have excluded from the Ordinance all homes receiving less than six patients—in other words, the whole host of little homes where the worst abuses are apt to creep in. Moreover, they have not stipulated that the head of a nursing home should be a trained and registered nurse—a most important point. Both hospitals and nurses appear to be suffering from Ordinances "flung on to the profession without warning and without consultation." The hard-and-fast rule as regards nurses' working hours imposed by a non-professional body, has caused great dislocation in some institutions. It is announced that the term of training at the Johannesburg Hospital has been raised from three to four years on account of the high percentage of failures among the pupil nurses at their final examination. It is argued that under the Nurses' Relief Ordinance sufficient time is not allowed the students during a three years' course to master their subjects.

The problem of Irish nursing is undoubtedly complicated by the fact that public bodies are apt to adopt a controversial attitude for or against the employment of nuns as nurses. At the present time an acute state of feeling has been produced by the proposal of the Dublin Corporation to replace in the Corporation Sanatorium the lay matron, lately resigned, and the other nurses by members of a religious order. It is urged by Irish nurses, who protest against this step, that if the process goes much further only Catholic nurses will be able to find posts in Ireland; Protestant

**ROUND THE HOSPITALS**—*(continued)*.

nurses will have either to enter a religious order or else to emigrate. The points which really need thinking out are : (1) whether nuns receive adequate training in the duties they are called on to perform in the wards; (2) whether their ministrations are preferred by the average patient to those of the average nurse. If Irish nuns are more popular than lay-women the lay Irish nurses must disarm criticism by studying to acquire the qualities so much liked in their rivals.

WE have received a letter from a correspondent who states she did fifteen hours' work a day during the war, and signs herself "Truth," calling attention to the long hours nurses are called on to work in certain rate-supported institutions. We are entirely in accord with her remark that sweated labour in so great a profession as nursing is a disgrace; that twelve hours on night duty, without relief, in fever or general wards is cruelly excessive; and that it is fully time that all backward institutions should be set to rights through the Ministry of Health. We must allow that new department of the State time to get into its stride, but we trust "Truth" will see her hopes realised before her "voyage into the beyond." We cordially appreciate her thanks for our "clear statements of facts in the past." She is right in fixing her hopes on the College of Nursing as the basis on which true reforms in the future will be built.

A MEETING of thanks to the war workers, voluntary and professional, at the Moor Park Hospital, Preston, was held at the Preston Guildhall on January 15. Nearly 600 certificates were presented by Colonel C. J. Trimble, C.M.G., who referred to some details of the work, especially the transport of wounded men. In addition to the general presentation of certificates, cheques were handed to Mrs. Howard (matron), Mrs. Pickles and Mr. G. D. Adams (Hon. Sec.), The Mayor, Councillor T. Parkinson, presided over the meeting and a concert which followed.

THE movement for developing the profession of nursing among native Indian women has been at a standstill during the war, but at a meeting held lately at the Countess of Minto's house in Lancaster Gate, Lady Carmichael made a lucid speech in which she poured into it fresh life. She explained from her own personal knowledge the great need for Indian women as nurses. In 1912 she visited all the hospitals of the Presidency and found no nurses, except in Calcutta. Trained matrons have been appointed at four hospitals, but the cost of bringing out English sisters is prohibitive. Even when trained, Indian nurses are at a great disadvantage, owing to the strong social prejudice in India against the adoption of this career. The remedy is to found a hostel where trained Indian nurses can live and pursue their calling under favourable conditions. The sum of £562 has already been contributed, and the appeal is strongly supported by the best Indian as well as Anglo-Indian authorities.

BIRMINGHAM is making an interesting effort to apply its war organisation to the need for hospital requisites in Central Europe. Small depôts are already at work, and a meeting has been held to arrange the preliminaries of a central depôt. Articles will probably be sent in bales direct from Birmingham to Vienna, and the Lady Mayoress (Mrs. W. A. Cadbury), who is president of the fund, suggested the possibility of a Birmingham unit going out to Vienna. The workers will probably concentrate at first on clothing and bedding for Vienna, Hungary and Poland. Reports from Vienna and the typhus-ridden districts of Poland were presented to the meeting. A very enthusiastic beginning has been made of a scheme which will be valuable in reviving the splendid spirit of the days of war, and should do a great deal to keep alive the consciousness of the needs of our own hospitals. The example of Birmingham might well be followed in many other places where enthusiastic war-time workers are lamenting the absence of opportunities for the kind of service and co-operation shown—and enjoyed—while the war was on.

A DAILY paper is responsible for a report from New York that the transfusion of blood has become such a frequent process at the Flower Hospital, New York, that a standard rate of £5 per pint has been established by the subjects willing to co-operate with the surgeon. Lately the price has been raised to £7, and a case is recorded where the subject at the moment of the operation demanded £11. He overreached himself by his avarice, however, for one of the nurses in attendance, Sister Jedlica, proffered her services free of charge, and, as other nurses have promised to follow suit as required, this curious mode of earning a living has come to an abrupt end. All the same, we are not quite sure that this is the right way out of the difficulty.

A WRITER in the *Irish Independent* exposes the backward condition of nursing affairs in Ireland as evidenced in the lack of adequate training centres for nurses among the Irish Union infirmaries. The present system appears to be very curious. "A nurse is appointed to each ward, and if she is ill or goes on a holiday a substitute is appointed at special rates of pay." The annual yearly cost of these substitutes, who are spoken of as "rough, incompetent, and often inhuman or bad-tempered," is extremely large, and the writer contends that the introduction of probationers instead would result in economy. Something more, however, is required in a training-school than the engagement of probationers, and nothing but such a radical alteration in Irish Unions as should sweep away together with the Guardians countless hoary abuses, would make them a fit place for training nurses. The time would seem, however, to be approaching when we may look for the regeneration of the Irish Union Infirmary under the Ministry of Health.

